# Intralesional Corticosteroid Therapy for Isolated Immunoglobulin G4-Related Esophageal Involvement: A Novel Approach to a Rare Condition

**DOI:** 10.14309/crj.0000000000002009

**Published:** 2026-02-23

**Authors:** Balkeess Alhanaktah, Emad Chishti, Nathan R. Shelman, Bahaaeldeen Ismail

**Affiliations:** 1 Department of Internal Medicine, Prince Hamza Hospital, Amman, Jordan; 2 Department of Internal Medicine, Johns Hopkins University School of Medicine, Baltimore, MD; 3 Department of Pathology and Laboratory Medicine, University of Kentucky, Lexington, KY; 4 Department of Digestive Diseases and Nutrition, University of Kentucky, Lexington, KY

**Keywords:** dysphagia, IgG4 disease, esophageal stricture, intralesional steroids

## Abstract

Immunoglobulin G4-related disease is a chronic, systemic immune-mediated condition. Esophageal involvement is considered rare and often poses major diagnostic and therapeutic challenges. We present an 81-year-old male patient with isolated esophageal immunoglobulin G4-related disease with extended clinical and endoscopic follow-up for 6 years, who had poor tolerance to systemic and topical steroids. The patient was successfully treated using a combination of intralesional steroids and serial balloon dilations spaced at long intervals. This approach, which has not been previously reported in this condition, resulted in prolonged clinical stability over an extended period.

## INTRODUCTION

Immunoglobulin G4-related disease (IgG4-RD) is a chronic fibroinflammatory condition mediated by a dysregulated immune response involving lymphocytes and plasma cells eventually leading to fibrosis.^1^ Characteristic histology includes IgG4-rich lymphoplasmacytic infiltrate, storiform fibrosis, and obliterative phlebitis.^2^

The pancreato-hepatobiliary tract is the most commonly involved region in gastrointestinal IgG4-RD, whereas luminal gastrointestinal involvement is rare. More than 20 esophageal IgG4-RD cases are reported, and while presentation and diagnosis are described, treatment approaches and disease course remain unclear, largely because of the limited follow-up time in the available cases. We report a case of esophageal IgG4-RD with six-year follow-up, successfully managed with multiple therapies, including the novel use of intralesional steroids.

## CASE REPORT

An 81-year-old man presented with progressive dysphagia to solids and regurgitation over 2–3 years, worsening rapidly in the 6 months before his referral. Medical history included hypertension and type 2 diabetes. He denied smoking or alcohol use. A modified barium swallow showed cervical osteophytes without functional impact. Laboratory results showed normal blood counts and metabolic panel.

**Figure 1.**
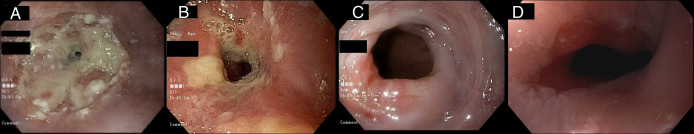
Endoscopic appearance of the stricture at initial presentation a 6 mm × 1 cm inflamed stricture at the gastroesophageal junction, with no other mucosal or intraluminal findings noted throughout the esophagus (A). (B) After serial endoscopic dilation alone at 9 months, (C) after serial endoscopic dilation with intralesional steroid injection at 19 months, and (D) most recent esophagogastroduodenoscopy five years from initial presentation.

**Figure 2.**
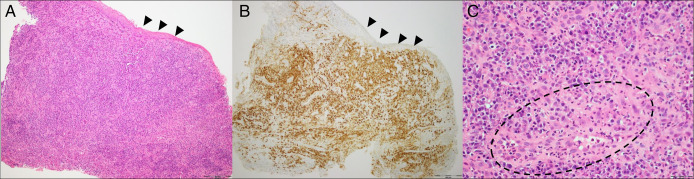
Histopathological findings from stricture biopsy at time of diagnosis. (A) Squamous mucosa [black arrowheads] undermined by dense infiltrate of plasma cells, with stromal sclerotic change (H&E, 100×). (B) Squamous mucosa [black arrowheads] undermined by dense infiltrate of plasma cells [IgG4 positive, brown chromagen], with stromal sclerotic change (IgG4 IHC, 100×). (C) High power field [1 HPF] demonstrating infiltration of plasma cells and area of early sclerotic change (interrupted line) with associated small vessel (asterisk) (H&E, 400×). H&E, hematoxylin and eosin; IgG4, immunoglobulin G4; IHC, immunohistochemistry.

He then underwent his first esophagogastroduodenoscopy (EGD) that revealed a 6 mm × 1 cm inflamed stricture at the gastroesophageal junction, with no other abnormalities (Figure 1). Dilation was deferred because of inflammation, and omeprazole 40 mg was increased to twice daily for suspected gastroesophageal reflux disease. The stricture biopsies revealed >100 IgG4-positive plasma cells per high-power field and a 40% IgG4-to-total plasma cell ratio, suggesting IgG4-RD (Figure 2), despite normal serum IgG4 and negative antinuclear antibody. Chest computed tomography and magnetic resonance cholangiopancreatography showed no evidence of IgG4-RD in other organs.

Four weeks later, EGD was repeated showing persistent inflammatory stricturing and underwent through-the-scope balloon dilation to 8 mm. Serial dilation every 1–2 weeks was advised to reach 15–18 mm, but the patient opted for less frequent sessions. Two months later, EGD showed persistent 8 mm stricture that was dilated to 12 mm. Oral prednisone 40 mg daily was initiated then and tapered over 3 months, which was associated with worsening hypertension and hyperglycemia. Six months later, the stricture regressed to 8 mm and was dilated to 11 mm with a 40 mg triamcinolone injection. A trial of compounded viscous budesonide (2 mg BID) was started but discontinued after 2 months because of lack of improvement and patient's concerns about side effects. Over the next 10 months, he underwent 3 additional EGDs with serial dilations and triamcinolone injections, eventually reaching 15 mm despite 4–5-month intervals.

He was subsequently monitored clinically, satisfied with his swallowing, and declined further EGDs. He remained asymptomatic until dysphagia reoccurred after 4 years. EGD revealed a 9 mm stricture (Figure 1), dilated to 13.5 mm with triamcinolone injection. He remains well under clinical follow-up.

## DISCUSSION

Although esophageal IgG4-RD has been reported, its natural history and treatment lines remain unclear. It is often omitted from expert reviews on immune-mediated esophageal conditions and strictures, highlighting the need for enhancing clinicians' awareness of this rare but treatable disorder.^3^

The typical presentation is progressive solid food dysphagia and variable-length inflammatory stricturing, as seen in our case. Esophageal involvement may also manifest as pseudotumors or mimic achalasia or esophagitis dissecans superficialis.^4–6^ Our patient exhibited a localized gastroesophageal junction stricture, with characteristic histopathology of dense lymphoplasmacytic infiltrates and an IgG4-to-IgG plasma cell ratio exceeding the reported cut-off of 40%–50%.^7^ Demographically, our case aligns with the literature, which shows a male predominance and peak incidence above age 50.^8^ Notably, only 2 cases have been documented in patients older than 80 years, matching our patient's age.^9^ While a few cases have demonstrated gastric, hepatic, or pulmonary involvement, our patient had no extra-esophageal disease and had normal serum IgG4 levels.^10,11^

Treatment options previously included surgery when malignancy or achalasia were suspected.^8,12^ This is generally unnecessary once this benign condition is correctly identified. Most cases report a combination of endoscopic dilation and medical therapy.^13,14^ Medical management as summarized in (Table 1) is largely extrapolated from other IgG4-RD, particularly autoimmune pancreatitis, where systemic corticosteroids are first-line.^4^ While effective in some, long-term steroid use is limited by side effects, as in our patient. Steroid-sparing agents such as rituximab and mycophenolate mofetil have shown partial benefit in isolated reports, but were not used in our patient because of limited evidence and patient's adverse events concerns.^6,8^

**Table 1.** Summary of previously reported IgG4-related esophagitis cases treated with immunosuppression

Study	Age	Sex	Presenting symptoms and duration before diagnosis	Pattern of esophageal involvement	Location of the esophageal lesion	Serum IgG4 levels	Involvement of other organs	Medical treatments used (in order)	Endoscopic treatments used (in order)	Surgical interventions	Outcome	Estimated follow-up duration
Dumas-Campagna et al,^6^ 2014	63	Female	Progressive odynophagia, dysphagia and weight loss (10 yr)	Circumferential ulcerative esophagitis with stenosis and dissecans superficialis appearance	Three distinct stenoses throughout the esophagus	NA.	None	Prednisone; oral mercaptopurine; oral mycophenolate mofetil; oral cyclosporine; infliximab; adalimumab; rituximab	None	None	Minimal response to prednisolone and rituximab but no significant improvement with other modalities	3 yr
Oh et al,^15^ 2015	33	Male	Progressive dysphagia and weight loss (1 yr)	Stricture and neck mass surrounding the lower cervical esophagus	Cervical esophagus just below the UES	Elevated	Isolated	Prednisolone (40 mg/d tapering by 10 mg/2 wk, then maintenance 5 mg/d)	None	None	Improved (able to tolerate solids)	11 mo
Yang et al,^10^ 2015	60	Male	Occasional acid reflux (workup was initiated because of esophageal masses on imaging)	Hard, fixed mass, normal overlying mucosa	Lower esophagus	Elevated	Stomach (ulcer) and liver (masses)	Steroids (no further details)	None	None	Stationary 3 mo after steroids (neither regression nor progression of the esophageal mass)	3 mo
Obiorah et al,^8^ 2017	63	Male	Dysphagia (20 yr)	Achalasia-like (distal esophagus dilation), along with esophagitis	Distal esophagus	NA.	None	Steroids (no further details)	None	Botox (for suspected achalasia)	Stationary	NA.
	47	Male	Dysphagia (6 yr)	Stricture with friable mucosa	NA.	Normal	None	Prednisolone initiated (0.6–1 mg/kg daily) then tapered and use maintenance mycophenolate mefotil	Multiple endoscopic dilations (before diagnosis)	None	Improved after prednisolone and mycophenolate mefotil	NA.
	79	Female	Dysphagia (14 yr)	Stricture with friable mucosa	NA.	Normal	None	Steroids (no further details), then mycophenolate mefotil	Multiple endoscopic dilations	None	Improved after prednisolone and mycophenolate mefotil	NA.
Sharma et al,^16^ 2018	18	Male	Dysphagia and weight loss (2 yr)	Stricture	Distal 7–8 cm	Elevated	None	Steroids (no further details)	Endoscopic dilation once	Esophagectomy	Uneventful postoperative period	NA.
Jang et al,^17^ 2019	56	Male	Dysphagia to solids and weight loss (for 3 yr)	Stricture and mucosal abrasion	At 32 cm (from incisors)	Normal	None	Prednisolone (30 mg slow taper	None	Ivor-Lewis esophagectomy (patient preferred more definitive therapy)	Uneventful postoperative course and improved after prednisolone	11 mo
Kaneshiro et al,^9^ 2022	87	Female	Dysphagia to solids (1 yr)	Stricture	At 27 cm (from incisors)	NA.	None	Swallowed fluticasone inhaler	Twelve endoscopic dilations	None	Minimal symptomatic relief, but inflammation worsened on following biopsy (3 mo after fluticasone)	24 mo
	65	Male	Dysphagia to solids and liquids and odynophagia	Diffuse narrowing with dissecans superficialis appearance. Then new focal stricture on follow up	Focal stricture at 20 cm (from incisors)	NA.	None	Oral Corticosteroids (no further details)	None	Esophago-gastrectomy (for eventual diagnosis of squamous cell carcinoma, proximal esophagus)	No response, after 6 mo of steroids	6 mo
Poole et al,^14^ 2022	47	Male	Impacted food bolus obstruction, progressive dysphagia and weight loss (6 mo)	Firm impassable stricture with mild inflammation	Stricture at 30 cm (from incisors) and circumferential narrowing from 30 cm to GEJ.	Elevated	None	Intravenous Hydrocortisone then prednisolone and azathioprine maintenance	Endoscopic dilation to 14 mm	VATS core biopsy (because of hypoechoic esophageal wall thickening)	Symptomatic improvement; recurrence upon weaning prednisolone; sustained improvement on azathioprine monotherapy	2.5 yr
Correia et al,^13^ 2023	30	Female	Dysphagia and odynophagia (yr)	Fibrous ring/membrane	Proximal esophagus	Normal	None	Topical fluticasone	Endoscopic dilation twice (complicated by perforation)	None	No significant improvement after 8 wk of fluticasone	10 mo

EGD, esophagogastroduodenoscopy; GEJ, gastroesophageal junction; IgG4, immunoglobulin G4; NA, not available; UES, upper esophageal sphincter; VATS, video-assisted thoracoscopic surgery.

Unlike pancreatic disease, luminal involvement is endoscopically accessible, enabling intralesional therapy. A similar approach has been applied in periorbital IgG4-RD, where local steroid injections proved effective.^18^ Several studies, including randomized trials, have shown that intralesional steroid injections during dilation prolong intervals between sessions in benign esophageal strictures, with a favorable safety profile.^3,19^ Though not previously reported in esophageal IgG4-RD, we found this a rational option to our patient given the immune-mediated disease nature and his localized stricturing. He tolerated repeated triamcinolone injections without adverse effects and experienced clinical and endoscopic improvement despite infrequent dilation sessions.

The disease's natural history remains poorly defined due to limited follow-up in most reports. Only 6 published cases included follow-up beyond 1 year after diagnosis, among them, 2 were followed after complete endoscopic or surgical disease resection, neither requiring further treatment.^5,20^ The others received medical therapies with mixed outcomes: one responded adequately and transitioned from corticosteroids to azathioprine monotherapy, while the others showed limited response (one to swallowed fluticasone, the other to various immunosuppressants).^6,9,14^ Our patient was followed clinically and endoscopically for 6 years, representing the longest follow-up duration reported to our knowledge. Notably, 4 of those years were without medical or endoscopic intervention, during which he experienced an unexpected slow symptoms progression. This variability may reflect disease behavior heterogeneity, with individual factors such as diet and symptom perception possibly contributing.^21^

Esophageal IgG4-RD should be considered in benign strictures, even without extra-esophageal involvement. Biopsies and appropriate staining are essential, especially with plasma cell infiltrates. Intralesional steroids appear safe and potentially effective. A watch-and-wait approach seems reasonable in those with indolent off-treatment course.

## DISCLOSURES

Author contributions: B. Alhanaktah and B. Ismail led the conception and design of the manuscript. Clinical data collection was performed by E. Chishti and NR Shelman. B. Alhanaktah and B. Ismail wrote the first draft of the manuscript, and all authors provided comments on this version. All authors read and approved the final manuscript. B. Ismail is the article guarantor.

Financial disclosure: None to report.

Informed consent was obtained for this case report.

## ABBREVIATIONS:

BID: Twice Daily
EGD: Esophagogastroduodenoscopy
GEJ: Gastroesophageal Junction
IgG4: Immunoglobulin G4;IgG4-RD
Immunoglobulin G4–Related Disease;IHC: Immunohistochemistry
UES: Upper Esophageal Sphincter
VATS: Video-Assisted Thoracoscopic Surgery
